# PReS-FINAL-2125: A Japanese girl with childhood-onset anti-Ku antibody positive generalized morphea-myositis overlap syndrome

**DOI:** 10.1186/1546-0096-11-S2-P137

**Published:** 2013-12-05

**Authors:** T Kishi, T Miyamae, A Seki, M Shioda, K Ishigaki, R Morimoto, N Ishiguro, Y Hamaguchi, M Fujimoto, Y Kawaguchi, H Yamanaka, S Nagata

**Affiliations:** 1 Institute of Rheumatology, Tokyo Women's Medical University, Tokyo, Japan; 2 Department of Pediatrics, Tokyo Women's Medical University, Tokyo, Japan; 3 Department of Dermatology, Tokyo Women's Medical University, Tokyo, Japan; 4 Department of Dermatology, Kanazawa University Graduate School of Medical Science, Ishikawa, Japan

## Introduction

Anti-Ku antibodies are autoantibodies against the P70/80 DNA-PK activated factor. These antibodies were identified in patients with scleroderma-polymyositis overlap syndrome by Mimori et al., and have subsequently been identified in approximately 1% of children with overlap syndromes.

## Objectives

We report the case of a Japanese child with anti-Ku antibody positive overlap syndrome.

## Methods

We retrospectively explore the difference between childhood anti-Ku positive syndromes, juvenile dermatomyositis and adult onset anti-Ku positive syndromes.

## Results

The patient, a 16-year-old Japanese girl, first developed symptoms at the age of 7. Her initial symptoms consisted of multiple brownish plaques on her left forearm that gradually extended to her upper arm, back, and left thigh. She underwent a skin biopsy at the age of 8 that revealed generalized morphea(GM). Laboratory findings included positive anti-nuclear antibody (1:1,280) and elevated serum creatine kinase (CK, 1,249 U/L) even though she lacked clinical evidence of myositis, myocardial failure or muscular dystrophy. Repeated skin biopsy at the age of 14 revealed lymphocytic infiltrations around vessels and thickened collagen bundles in the dermis. Although she still lacked clinical signs of muscular involvement, MRI demonstrated findings consistent with myositis and bilateral thigh atrophy. Furthermore, serum anti-Ku antibody was detected by immunoprecipitation assay. She was thus diagnosed with generalized morphea-polymyositis overlap syndrome. She was treated with methylprednisolone pulse therapy followed by oral glucocorticosteroids which resulted in a gradual decrease in serum CK levels.

Three patients with adult onset anti-Ku antibody syndrome also presented to our institute. All three patients were female, and the average age of onset was 36.0 years old (range 30-46)(Table 1). Each patient was treated with glucocorticosteroids, but immunosuppresnts were ultimately required to suppress disease activity in all three cases.

**Table 1:** 

	Diagnosis	Initial symptom	**Age at onset**, Gender	Observation period	Raynaud phenomenon	Sclerodactyly	Myositis	Organ involvement	CK(U/l)/ Ald(U/l)	ANA
clild onset	GM/PM	Multiple skin lesions	7y Female	8y6m	-	+	+	-	1,249/ 20.9	1:1,280

adult onset	DM	Muscle weakness,Weight loss	30y Female	2y2m	+	-	+	IP	4,699/ 53.8	1:2,560

	SSc/PM	Muscle weakness, Joint pain	32y Female	12y0m	+	+	+	ED	1,078/ 16.6	1:10,240

	SSc/PM	Arthritis	46y Female	4y10m	+	+	+	IP,ED, Arthritis	1,825/ 30.9	1:640

## Conclusion

Our pediatric patient represents one of the youngest reported cases of anti-Ku antibody positive overlap syndrome. Consistent with previously reported cases, she had remarkably high levels of serum CK despite a lack of muscle weakness. The child had a slowly progressive clinical course with no organ involvement. Further reports are required to fully determine the characteristics of childhood-onset anti-Ku antibody positive overlap syndrome.

## Disclosure of interest

None declared.

